# Corticospinal Adaptation to Short-Term Horizontal Balance Perturbation Training

**DOI:** 10.3390/brainsci13081209

**Published:** 2023-08-15

**Authors:** Nijia Hu, Jarmo M. Piirainen, Dawson J. Kidgell, Simon Walker, Janne Avela

**Affiliations:** 1 NeuroMuscular Research Center, Faculty of Sport and Health Sciences, University of Jyväskylä, FI-40014 Jyväskylä, Finland; simon.walker@jyu.fi (S.W.); janne.avela@jyu.fi (J.A.); 2 Sports Technology Program, Faculty of Sport and Health Sciences, University of Jyväskylä, FI-88610 Vuokatti, Finland; jarmo.m.piirainen@jyu.fi; 3 School of Primary and Allied Health Care, Department of Physiotherapy, Monash University, Frankston P.O. Box 527, Australia; dawson.kidgell@monash.edu

**Keywords:** balance control, motor learning, transcranial magnetic stimulation, H-reflex, automaticity

## Abstract

Sensorimotor training and strength training can improve balance control. Currently, little is known about how repeated balance perturbation training affects balance performance and its neural mechanisms. This study investigated corticospinal adaptation assessed by transcranial magnetic stimulation (TMS) and Hoffman-reflex (H-reflex) measurements during balance perturbation induced by perturbation training. Fourteen subjects completed three perturbation sessions (PS1, PS2, and PS3). The perturbation system operated at 0.25 m/s, accelerating at 2.5 m/s^2^ over a 0.3 m displacement in anterior and posterior directions. Subjects were trained by over 200 perturbations in PS2. In PS1 and PS3, TMS and electrical stimulation elicited motor evoked potentials (MEP) and H-reflexes in the right leg soleus muscle, at standing rest and two time points (40 ms and 140 ms) after perturbation. Body sway was assessed using the displacement and velocity of the center of pressure (COP), which showed a decrease in PS3. No significant changes were observed in MEP or H-reflex between sessions. Nevertheless, Δ MEP at 40 ms demonstrated a positive correlation with Δ COP, while Δ H-reflex at 40 ms demonstrated a negative correlation with Δ COP. Balance perturbation training led to less body sway and a potential increase in spinal-level involvement, indicating that movement automaticity may be suggested after perturbation training.

## 1. Introduction

Balance control is a fundamental motor skill that requires rapid adaptation to a dynamically changing environment (e.g., balance perturbation tasks) [1]. Especially in dynamic tasks, balance control involves proprioceptive, somatosensory, and vestibular loops, which are related to neuronal activity in the brain stem, cerebellum, and motor cortex [2,3]. It has been determined that inherent muscle activity and the action of reflex loops contribute to maintaining standing balance during balance perturbation tasks [4,5]. Muscle activity recorded by electromyography (EMG) in lower limb muscles, such as the soleus and tibialis anterior, exhibits a stereotypical pattern referred to as short-latency response (SLR), medium-latency response (MLR), and long-latency response (LLR) following ankle movement subsequent to the disturbance of a standing position [6,7]. Although there have been different opinions regarding latencies and the mechanisms of these responses, SLR is generally considered a monosynaptic reflex and can enhance muscle stiffness [8,9]. Alternatively, MLR is more likely due to group-II afferent involvement [10] and is likely subcortical in origin, while LLR is associated with transcortical loops [11,12].

Several studies have examined how sensorimotor training [12], explosive strength training [13], and cognitive training [14] can improve balance control. In this regard, neural adaptation at both spinal and supraspinal levels has been demonstrated following training [12,13,14,15,16,17,18]. The Hoffman reflex (H-reflex) is a commonly used method to evaluate neural excitability at the spinal level. Correia et al. [19] reviewed that there were only a few studies that had used H-reflex in fast movement tasks. Even though H-reflex changes (i.e., spinal circuit excitability) occur before voluntary reactions, these reflex reactions may be more related to movement force rather than other factors, such as speed. On the other hand, transcranial magnetic stimulation (TMS) is a noninvasive method to investigate corticospinal excitability in human sport and training [20]. Taube et al. [18] showed that neural adaptation occurred more at the cortical level than at the spinal level through TMS-evoked motor evoked potentials (MEP) before and after balance training. Reduced MEPs and TMS-conditioned H-reflexes in the LLR but not SLR were observed during a balance perturbation task. Therefore, neural adaptation seems to occur at both spinal and supraspinal levels after balance training, depending on the timing of interest after the perturbation. Thus, responses in the SLR and LLR have been shown to differ.

Since balance control is a motor skill, balance training can also be considered motor skill learning acquisition. Rosenkranz et al. [21] showed that short-term (five sessions) training demonstrated synaptogenesis, and, thus, improved corticospinal plasticity. The activity of the primary motor cortex has been demonstrated in the early learning phase of a static balance task [22]. However, the study of Prsa et al. [23] indicated that motor skill training in a laboratory setting (e.g., moving an arm to a required target) is simpler than in ‘real-life’ situations, where tasks can be more complex and require conscious effort to complete. In other words, simple motor training tasks in the laboratory may be acquired quickly, even after a few trials, and in an unconscious manner. This is often referred to as ‘implicit learning,’ to distinguish it from ‘motor skill acquisition’ [24]. When using balance perturbation as a motor task, hundreds of perturbations are usually involved in one experiment, which may already induce the ‘adaptation’ of the central nervous system. Therefore, it is important to clarify how balance control ability improves and adapts to short-term repeated perturbation tasks.

When perturbation tasks are more challenging and unfamiliar (i.e., higher perturbation amplitude and velocity), well-coordinated and higher muscle activities are needed to maintain body balance. Subsequently, high voluntary muscle activation after LLR has been shown in our previous study [25]. However, this important time window for muscle activity has not been frequently studied previously. This voluntary muscle activity may be related to a stronger contribution of cortical drive to maintain body position/restore balance during challenging perturbation tasks [26,27,28]. Currently, little is known about how repeated balance training affects balance performance and voluntary activation after LLR during balance perturbation tasks, or about the contribution of the spinal and supraspinal mechanisms behind this improvement. The main purpose of this study was to investigate whether short-term motor learning leads to performance improvement during ankle transitional perturbation. A secondary purpose was to determine the neural mechanism that might modulate the improvement in balance control ability. Specifically, this study investigated (1) the effect of high-amplitude short-term balance perturbation training on balance performance, and (2) corticospinal and spinal excitability at different time points after perturbation.

## 2. Materials and Methods

### 2.1. Subjects

Fourteen subjects volunteered to participate in the study (7 males and 7 females, age: 33 ± 5 years, height: 1.71 ± 0.93 m, weight: 72.8 ± 14.2 kg, and BMI: 24.6 ± 3.5). None of the subjects had any history of neuromuscular or orthopedic diseases and all subjects were informed about the procedures and gave written informed consent. Subjects were fully introduced to the protocol and they had the opportunity to withdraw from the study at any time. An ethical statement (1267/13.00.04.00/2021) was given by the ethics board of the University and the study was performed in conformity with the declaration of Helsinki (2013).

### 2.2. Experimental Design

**Figure 1:**
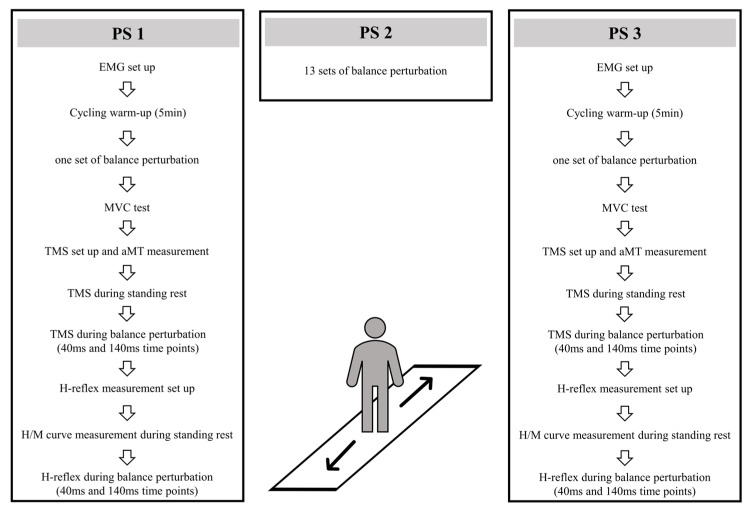
Experimental design in PS1, PS2, and PS3.

Three perturbation sessions (PS1, PS2, PS3) were conducted within 48 h intervals (see Figure 1). In PS1 and PS3, after EMG electrode setup and 5 min cycling warm-up (70 W) on a fitness ergometer (Monark, 282 E, Varberg, Sweden), 16 balance perturbations (1 set) were used to collect center-of-pressure (COP) and EMG activity data. After this, subjects were positioned in a custom-built ankle dynamometer (University of Jyväskylä, Jyväskylä, Finland) to measure the isometric maximal voluntary contraction force (MVC) of the right leg. The TMS coil was set on a hotspot and the active motor threshold (aMT) was tested when subjects sat in the ankle dynamometer. With a TMS coil attached to the head and held by the custom-built helmet [25], subjects moved carefully to the balance platform, and 10 TMS pulses were given to measure MEPs during standing rest. In the balance perturbation task with stimulation, MEPs were evoked at 40 ms and 140 ms time points after the onset of ankle movement during the balance perturbation in random order. H-reflexes were elicited at the same two time points and in random order. The stimulations were delivered during each balance perturbation (anterior and posterior directions), however, only MEPs and H-reflexes during posterior perturbations were analyzed. In PS2, 13 sets of balance perturbations (16 perturbations in each set) were given to subjects with 1–2 min rest between perturbation sets.

### 2.3. Isometric Maximal Voluntary Contraction

MVC was used to investigate possible muscle fatigue between sessions and to monitor muscle contraction levels during the identification of aMT (10% MVC). Subjects were positioned in a custom-built ankle dynamometer (University of Jyväskylä, Jyväskylä, Finland) to assess the MVC with the right foot on the pedal at 100° hip angle, 180° knee angle (leg fully extended) and 90° ankle angle. After the positioning procedure, the subject performed 5–7 submaximal plantarflexion trials to practice performance. MVC was performed at least three times at one-minute intervals and the highest force value was considered the MVC. If the last trial was >5% higher than the second-best, additional trials were performed until no further improvement was observed. The typical number of required maximum trials was 3–5. The force from the dynamometer pedal was measured by a strain gauge transducer sampled at 1 kHz in Spike2 (8.0) software (CED Ltd., Cambridge, UK), and the maximum MVC amplitude was analyzed.

### 2.4. Electromyography (EMG)

EMG was measured by bipolar electrodes (Blue Sensor, Ag/AgCl, 28 mm^2^, Ambu A/S, Ballerup, Denmark) placed 2 cm below the gastrocnemius on the line of the Achilles tendon for the soleus (SOL), tibialis anterior (TA), and gastrocnemius (GM) muscles according to SENIAM guidelines [29]. In our pilot TMS study, discomfort and muscle twitch were reported by some subjects at the 140 ms (voluntary activation) time point. To reduce the potential discomfort and tension caused by high-intensity stimulation, a pseudo-monopolar setup on the SOL was used in TMS measurements, as has been used previously [30,31]. The pseudo-monopolar setup provides a better representation of the electrical characteristics of the action potentials [32], resulting in a higher MEP amplitude compared to a bipolar arrangement with the same intensity of the stimulus. In addition, according to our practical experience, the shape of the MEP is more consistent with the pseudo-monopolar setup, which is important in dynamic tasks. A disadvantage of this electrode montage is that the signal-to-noise ratio can be compromised; however, this was not a problem in the current study. One electrode was placed 2 cm below the gastrocnemius on the line of the Achilles tendon and the reference electrode was placed on the tibia at the same level. The skin was shaved, carefully abraded with sandpaper, and cleaned with alcohol. The target skin impedance was less than 5 kΩ, and if this was not the case, skin preparation was repeated. All EMG data were collected using the Neurolog EMG system (CED Ltd., Cambridge, UK), with a gain of 1000. Data were band-passed filtered (15–500 Hz) and further collected using a CED 1401 A/D-converter (CED ltd., Cambridge, UK) and Spike 2 (8.0) software (CED Ltd., Cambridge, UK) with a sampling rate of 5 kHz.

### 2.5. Dynamic Balance Perturbations System

Balance perturbation tasks utilized a custom-built dynamic balance device (University of Jyväskylä, Jyväskylä, Finland) modified from Piirainen et al. [33] and Hu et al. [25]. The balance perturbation system operated at 0.25 m/s, accelerating at 2.5 m/s^2^ over a 0.3 m displacement. In balance perturbation tasks, 16 perturbations were performed in 1 set, with 8 anterior and 8 posterior perturbations. The order of the perturbation direction was the same in all sets. Two min rest periods were given after every perturbation set to minimize possible muscle fatigue [33]. Every perturbation was triggered when COP was below the ±5 mm level of the standing baseline for at least 1 s. This approach modified the triggering timing and ensured that the subject was always keeping the initial body position and not anticipating the upcoming perturbation. A fixation point was set on the wall 3 m from the subjects at eye level to stabilize the subjects’ visual attention during measurements.

During balance perturbation tasks, COP values were collected by a force plate embedded inside the balance platform. One strain gauge sensor was located in each of the four corners of the force plate (BT4 balance platform; HUR Labs, Tampere, Finland), and data were saved and analyzed using the Coachtech-feedback system (University of Jyväskylä, Jyväskylä, Finland). COP in the anterior-posterior direction was calculated using the formula: $\mathrm{COPy}=\left(\left(\mathrm{Flf}+\mathrm{Frf}\right)\times 0.26-\left(\mathrm{Flr}+\mathrm{Frr}\right)\times 0.26\right)/\left(\mathrm{Flf}+\mathrm{Frf}+\mathrm{Frr}+\mathrm{Flr}\right)$, where F is the force value from sensors (lf = left front, rf = right front, or = right rear, lr = left rear), and 0.26 (m) is the sensor distance from the middle line.

### 2.6. TMS Measurement Setup

TMS was delivered using a single-pulse Magstim 200^2^ stimulator with a double-cone coil (Magstim, Whitland, UK). A skin-tight (swimming) cap was placed on the head of the subject to increase friction between the coil and the scalp. The optimal TMS stimulation site for the right SOL was located on average 1 cm lateral (left) and 1 cm posterior to the cranial apex. Several stimulations were delivered to determine optimal coil placement and it was then marked by a marker pen on the cap. The aMT was the lowest stimulus intensity to elicit clear MEPs in three out of five stimulations from right ankle plantarflexion with 10% MVC [34,35]. After the confirmation of aMT, a second swimming cap with a hole in the middle of the vertex (Orca High Visibility Neoprene Swim Cap, Orca, Auckland, New Zealand) was placed over the coil to reduce the gap and relative movement between the coil and the head. Then, a custom-made helmet (modified from an ice-hockey helmet; CCM TACK 710 JK-K, CCM Hockey, Montreal, QC, Canada) was attached to the subject’s head with a chin strap. In the balance perturbation system, the TMS cable was placed on a conveyor adjacent to the safety belt conveyor on the roof and connected with the balance platform by a firm handle, which was the same as in our previous study [25]. Single-pulse TMS with a 110% intensity of aMT was delivered during standing rest and balance perturbation tasks to investigate corticospinal excitability, and 110% intensity would cause less discomfort than the higher level stimulations used in our pilot study.

During balance perturbation tasks, a constant delay (25 ms) between the platform control signal and the onset of ankle movement was reported in our previous study [25]. Therefore, 40 ms and 140 ms time points after ankle movement were defined as SLR and voluntary activation timing. MEP latency was calculated between the TMS pulse and MEP rising point during standing rest. Then, single-pulse TMS with 110% aMT was adjusted to elicit MEP arising at 40 ms and 140 ms time points.

### 2.7. H-Reflex Measurement Setup

For H-reflex measurements, subjects stood relaxed during the electrical stimulation setup. Electrical stimulation was administrated to the tibial nerve in the popliteal fossa. A cathode (1.5 cm × 1.5 cm) was placed over the tibial nerve, and an anode (5 cm × 8 cm) was placed above the patella. Rectangular stimulation pulses (DS7AH, Digitimer Ltd., Hertfordshire, UK) with a duration of 0.2 ms were delivered at 10 s intervals. Once the optimal site of stimulation was found, the site was marked by a marker pen, and an electrode (Blue Sensor, Ag/AgCl, 28 mm^2^, Ambu A/S, Ballerup, Denmark) was placed and strapped around the subject’s knee with an elastic band. An increasing intensity interval (1–5 mA) was chosen to measure the H/M recruitment curve, with at least 30 data points up to the maximal M-wave. The stimulus intensity was adjusted to 5% (±2%) of the maximum M-wave, which was used during balance perturbations to control the stimulation intensity in H-reflex measurements.

During balance perturbation tasks, the H-reflex was measured using the same protocol as in TMS trials. For the H-reflex, a successful trial from 1 perturbation set (16 perturbations) was defined as an M-wave response of 5% (±2%) of the maximal M-wave. The intensity of electrical stimulation was adjusted during perturbation trials to obtain at least five successful trials. If less than five successful H-reflex trials in a set were achieved, an extra perturbation set—four backward and four forward—was performed. For each perturbation task with stimulation, 5 successful H-reflexes were usually completed within 1 to 1.5 sets (16–24 perturbations).

### 2.8. Data and Statistical Analysis

**Figure 2:**
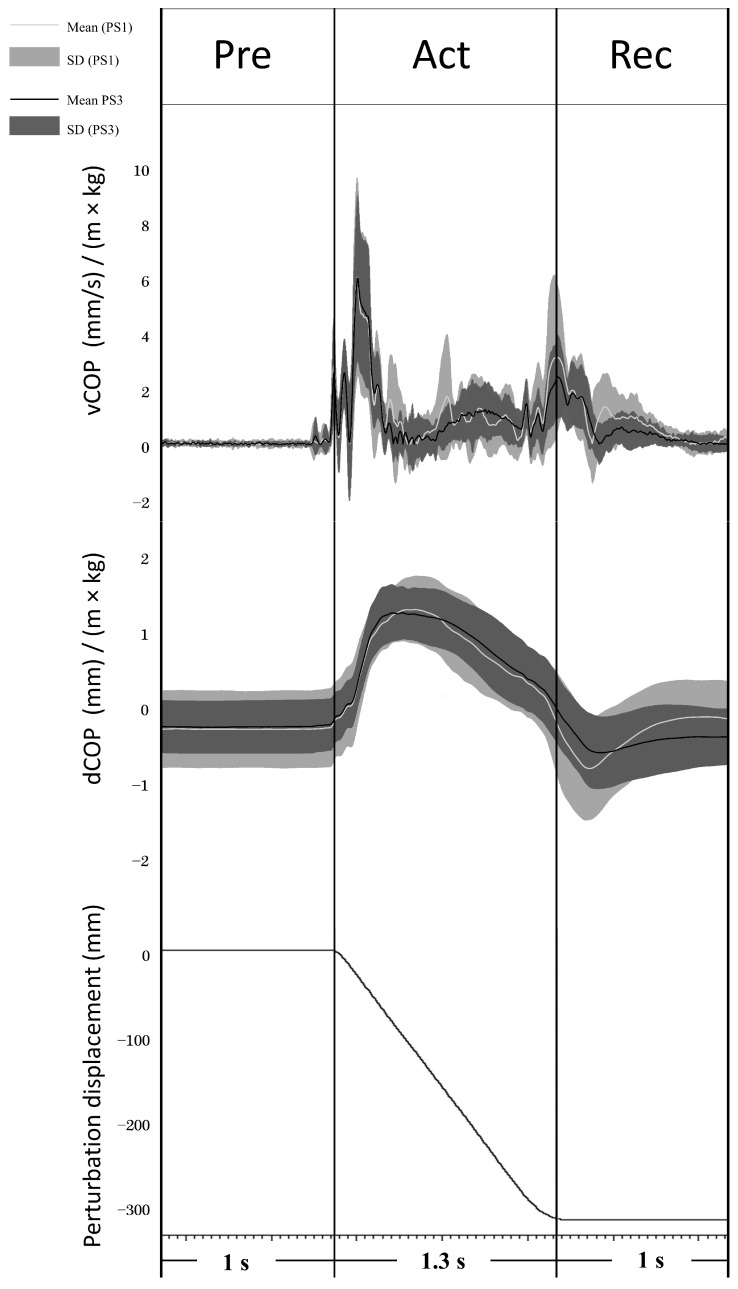
Average COP displacement and velocity (line) and their standard deviations (shadow) from 14 subjects in PS1 (light grey) and PS3 (black) are shown. The signal diagram (bottom part) shows the displacement of the balance perturbation (a negative value means the platform moved backward).

COP values were analyzed in perturbation trials without stimulations. The mean standard deviation of the COP displacement curve was calculated to evaluate the general body sway (COP_SD). Peak-to-peak COP displacement (dCOP) was analyzed over a time window of 1 s before platform movement (Preparation phase; Pre), during platform movement (Active phase; Act), and 1 s from the end of platform movement (Recovery phase; Rec) (see Figure 2). The COP velocity curve was calculated by differentiating the COP curve over 20 ms windows, and then the average COP velocity of the velocity curve (vCOP) was analyzed in the same time window as dCOP (see Figure 2). Both dCOP and vCOP were normalized by the individual subject’s height × weight (dCOP: mm/(m × kg); vCOP: (mm/s)/(m × kg)) according to the recommendation of Chiari et al. [36].

In the balance perturbation trials without stimulation, the average of all subjects’ full wave rectified EMG data from 100 ms before the perturbation onset to 400 ms after onset was analyzed. Furthermore, EMG activity was analyzed using root mean square (RMS) over 20 ms time windows from the perturbation onset (0 ms) to 180 ms after onset.

MEPs and the H-reflex were measured during standing rest and balance perturbation. During standing rest, a clear MEP was defined to start when EMG was above the mean + 2SD level recorded 100 ms before the TMS trigger and end when below the mean-2SD level [37]. The average MEP latency and the MEP duration were analyzed during the standing rest and then used to trigger TMS during the balance perturbation. Average MEP values were determined with peak-to-peak amplitude (in mV) from 10 TMS stimulations. 4Outliers were identified from the 10 trials (±2.5 SD) and removed before analysis [38]. In balance perturbation trials, the MEP amplitude from 7–8 trials was selected when the platform moved backward and was averaged after excluding outliers (±2.5 SD), which has been shown to provide good reliability in our previous study [25]. All MEPs were normalized to the peak-to-peak value of the maximal M-wave (M_MAX_) and presented as %M_MAX_ in the results. The H-reflex was determined as peak-to-peak amplitude and averaged from all successful trials (within 3–7% M_MAX_) in standing rest and balance perturbation tasks. The H-reflex was also normalized to the M_MAX_. The RMS of background EMG (BGemg) was also analyzed with a 30 ms window before TMS and H-reflex triggers and normalized to M_MAX_ (monopolar and bipolar).

In order to evaluate neural excitability changes between corticospinal and spinal levels, the MEP/H-reflex ratio (MEP/H ratio) was calculated. To explore the relationship between the changes in balance performance and corticospinal correlation from PS1 to PS3, the ∆MEP and ∆H-reflex and their correlation with ∆ dCOP were analyzed by Pearson product moment correlation. Delta values were calculated (i.e., MEP, H-reflex, and dCOP, respectively) using the formula: (value (PS3)—value (PS1))/value (PS1) × 100%.

The number of participants required was based on power calculations for the expected change in mean rectified MEPs (sEMG recordings from the soleus muscle during balance perturbation). By utilizing previous data from a similar experimental setup by Hu et al. [25], we estimated that 10 subjects in each condition would provide at least 80% power (with a 95% confidence interval) to detect a 15% difference in mean rectified MEPs. This calculation assumed an SD of 10–15% between time points, with a significance level set at *p* < 0.05 (two-tailed).

Statistical analyses were conducted using JASP (Version 0.17.1). Result visualizations were performed using Prism (V9, GraphPad Software, San Diego, CA, USA). Since the original data were not normally distributed, all variables of the MEP/H ratio were processed by log transformation before statistical analyses following Nielsen’s suggestion, which resulted in data being normally distributed, as assessed by Shapiro–Wilk W tests [39]. MVC, M_MAX_, and H_MAX_/M_MAX_ values were assessed by a paired *t*-test. Since dCOP and vCOP were analyzed by different time windows (i.e., 1 s for Pre and Rec phases but 1.3 s for the Act phase), between-session differences of dCOP, vCOP, and COP_SD were examined by paired *t*-test. To assess adaptation in corticospinal excitability during balance perturbations, MEPs, H-reflex, BGemg, and EMG activity were assessed by two-way (2 × 3) repeated-measures ANOVA with the factors SESSION (PS1 and PS3) and TIME (standing rest, 40 ms, 140 ms). When a significant F-value was observed, Mauchly’s test was used to evaluate sphericity, and where the assumption was valid, F-values were reported with sphericity-assumed degrees of freedom and df error (i.e., F (sphericity-assumed df, df error)). Effect sizes for the ANOVA main effects are reported as partial eta squared (η_p_^2^), where 0.02, 0.13, and 0.26 are considered small, medium, and large, respectively. If significance for TIME was revealed, Bonferroni post-hoc analysis was used for pairwise comparisons between levels (i.e., standing rest, 40 ms, 140 ms). Correlations between MEP amplitude, H-reflex amplitude, and EMG activity were analyzed by Pearson product-moment correlation tests. The significance level was set at *p* < 0.05 and all results were displayed as Mean ± SD in the text and figures.

## 3. Results

### 3.1. Balance Performance during Perturbation

dCOP (mm/(m × kg)) and vCOP ((mm/s)/(m × kg)) of Pre, Act, and Rec were analyzed to explore balance performance in AP direction before, during, and after the onset of balance platform movement, respectively (see Figure 2). Both dCOP and vCOP at all phases decreased significantly from PS1 to PS3 (see Table 1). COP_SD demonstrated a significant decrease at PS3 compared to PS1 (PS1: 0.16 ± 0.05; PS3: 0.13 ± 0.06, t _(13)_ = 2.741, *p* = 0.017).

### 3.2. Corticospinal Excitability during Perturbation

There was no significant difference demonstrated in M_MAX_ (PS1: 6.79 ± 1.33 mV, PS3: 6.64 ± 1.53 mV, t _(13)_ = 0.907, *p* = 0.381), H_MAX_/M_MAX_ (PS1: 48.5 ± 16.2%M_MAX_, PS3: 47.4% ± 18.2%M_MAX_, t _(13)_ = 0.279, *p* = 0.785), or MVC (PS1: 1756.9 ± 480.9 Nm, PS3: 1813.7 ± 480.9 Nm, t _(13)_ = −2.070, *p* = 0.059) between PS1 and PS3.

**Figure 3:**
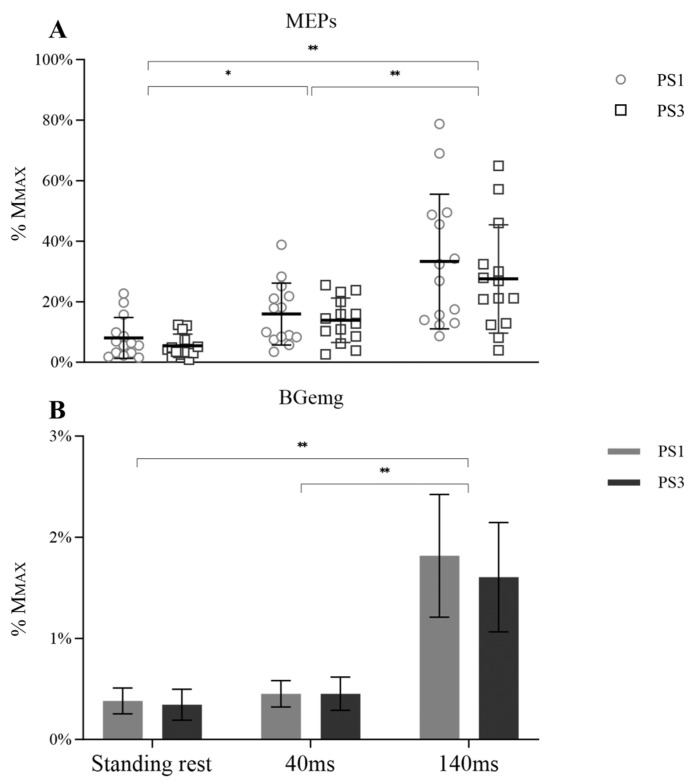
MEP (**A**) with BGemg (**B**) at three different times (standing rest, 40 ms, and 140 ms), in which symbols represent the MEP values of individual subjects and bar charts represent the BGemg activities from monopolar EMG setups. Significant differences between time points are marked with * (*p* < 0.05) and ** (*p* < 0.001).

A significant main effect for time was observed for MEP amplitude (Figure 3A, F _(1.118, 30.897)_ = 39.355, *p* < 0.001, η_p_^2^ = 0.602), but there was no main effect for session (F _(1, 26)_ = 0.817, *p* = 0.374, η_p_^2^ = 0.031) or session × time interaction (F _(1.118, 30.897)_ = 0.267, *p* = 0.650, η_p_^2^ = 0.010). However, significant differences over time were observed from 40 ms to 140 ms (*p* < 0.001). In addition, MEP amplitude during standing rest was lower than 40 ms (*p* = 0.012) and 140 ms (*p* < 0.001). BGemg values demonstrated an increase at 140 ms compared to other times (Figure 3B, *p* < 0.001).

**Figure 4:**
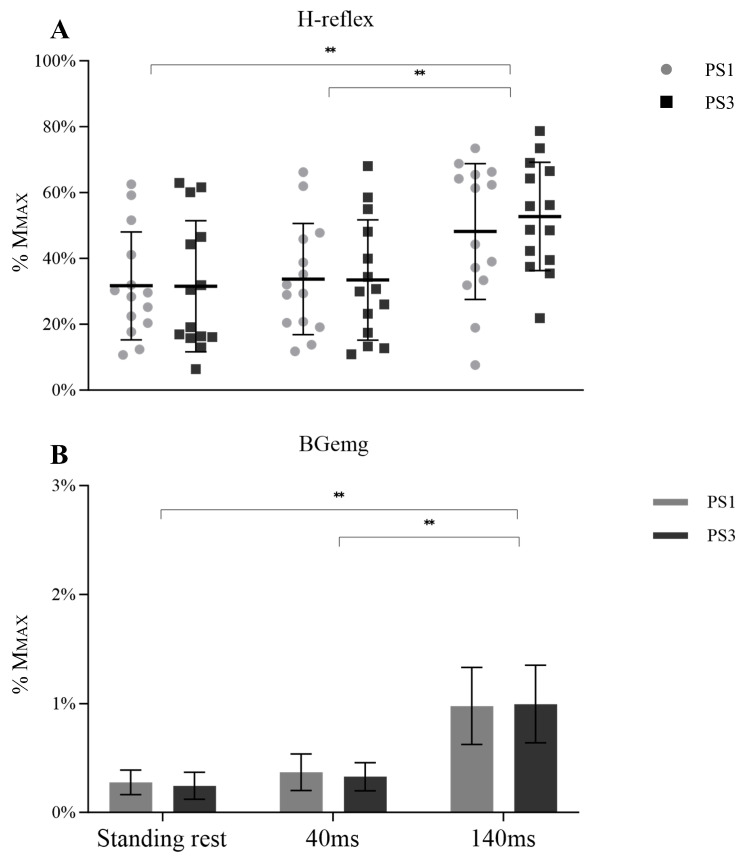
H-reflex (**A**) with BGemg (**B**) at three different times (standing rest, 40 ms, and 140 ms), in which symbols represent H-reflex values of individual subjects and bar charts represent the BGemg activities bipolar EMG setups. Significant differences between time are marked with “**” (*p* < 0.001).

Similarly, no between session difference was shown in the H-reflex (F _(1, 26)_ = 0.048, *p* = 0.828, η_p_^2^ = 0.002) or session × time interaction (F _(1.273, 33.099)_ = 0.638, *p* = 0.466, η_p_^2^ = 0.024). However, a significant main effect over time was observed (Figure 4A, F _(1.273, 33.099)_ = 36.269, *p* < 0.001, η_p_^2^ = 0.582). Post-hoc tests showed an increased H-reflex from 40 ms to 140 ms (*p* < 0.001). Plus, the H-reflex during the standing rest was lower than 140 ms (*p* < 0.001). BGemg values demonstrated an increase at 140 ms compared with the other times (Figure 4B, *p* < 0.001).

**Figure 5:**
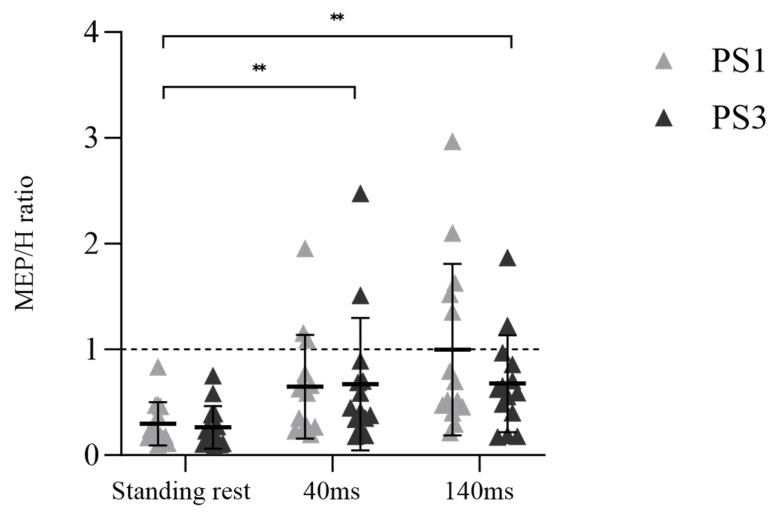
The ratio of MEP and H-reflex at three different times (standing rest, 40 ms, and 140 ms), in which symbols represent the values of individual subjects, while the mean values with standard deviation bars are also depicted. Significant differences between times are marked with “**” (*p* < 0.001).

A significant main effect for time was observed in the MEP/H ratio (Figure 5, F _(1.592, 41.397)_ = 37.174, *p* < 0.001, η_p_^2^ = 0.588), but there was no main effect for session (F _(1, 26)_ = 0.541, *p* = 0.469, η_p_^2^ = 0.020) or session × time interaction (F _(1.592, 41.397)_ = 0.704, *p* = 0.469, η_p_^2^ = 0.026). Post-hoc tests demonstrated an enhanced MEP/H ratio from standing rest to 40 ms (*p* < 0.001) and standing rest to 140 ms (*p* < 0.001).

### 3.3. Soleus Muscle Activity during Perturbation

**Figure 6:**
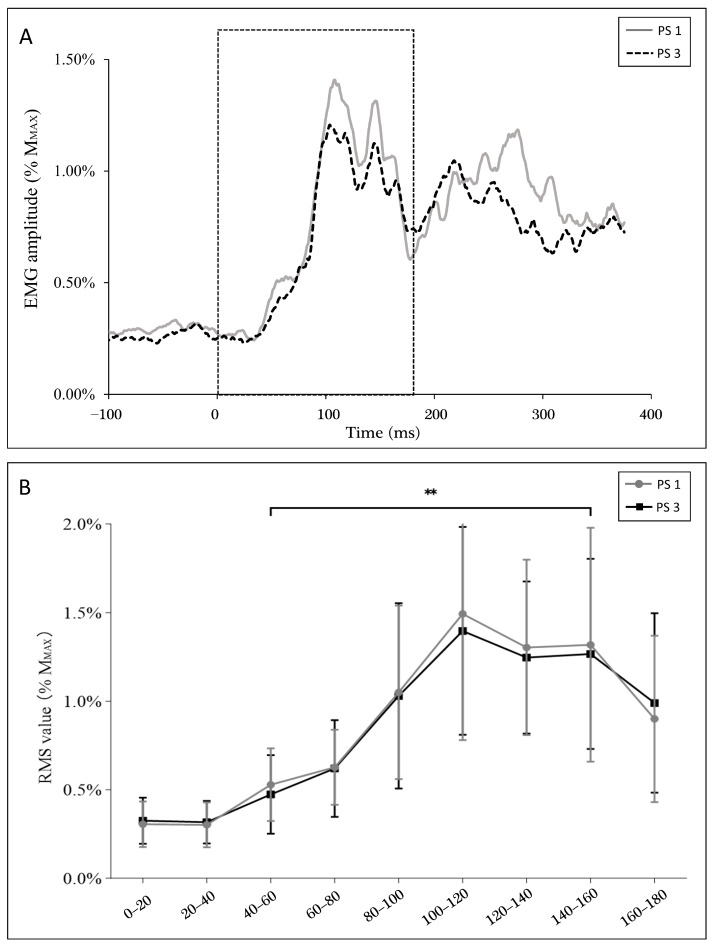
Average EMG activity data from all subjects in perturbation trials without stimulation (**A**). Data are normalized to M_MAX_. RMS of EMG activity (**B**) in every 20 ms window from ankle movement (0 ms) to 180 ms, as zoomed in from the dashed square of A. Statistical significance is denoted by “**” (*p* < 0.001). There are other significant differences between the time windows, which are not marked in the figure since this study focused on 40–60 ms and 140–60 ms windows.

Figure 6A shows soleus muscle activity during balance perturbation. Specifically, the RMS of EMG did not demonstrate a significant between-session difference in perturbation trials without stimulation. EMG activity was lower at the 40–60 ms window (PS1: 0.53 ± 0.20%M_MAX_, PS3: 0.47 ± 0.21%M_MAX_) when compared to the 140–160 ms window (PS1: 1.32 ± 0.64%M_MAX_, PS3: 1.27 ± 0.52%M_MAX_) (*p* < 0.001) (Figure 6B), where these windows match the timings of the stimulations.

Additionally, a positive correlation was observed between EMG activity (40 ms–60 ms) and H-reflex (r = 0.500, *p* = 0.007), but not MEP (r = 0.161, *p* = 0.412) at 40 ms. On the contrary, EMG activity showed a positive correlation with MEP (r = 0.501, *p* = 0.007), but not the H-reflex (r = 0.241, *p* = 0.218) at 140 ms.

### 3.4. Correlations between Corticospinal/Spinal Excitability and Balance Performance

**Figure 7:**
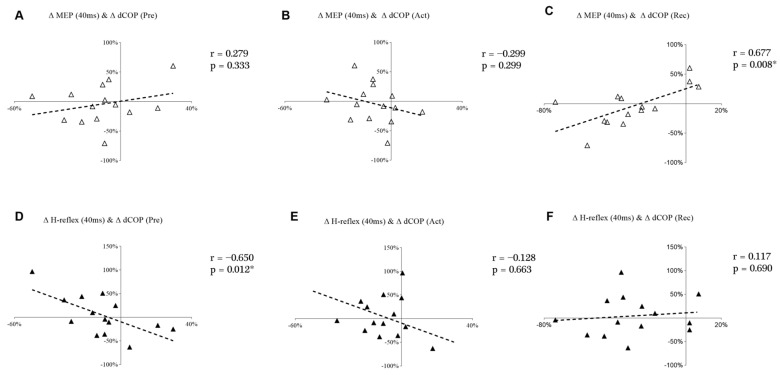
Scatter plots of Δ MEP with Δ dCOP at Pre (**A**), Act (**B**), and Rec (**C**); Scatter plots of Δ H-reflex with Δ dCOP at Pre (**D**), Act (**E**), and Rec (**F**). “*” is marked when *p* < 0.05.

Figure 7 shows the correlations between changes in the displacement of COP and MEP and the H-reflex at the 40 ms time point. Δ MEP at 40 ms demonstrated a significant and positive correlation with Δ dCOP in Rec (Figure 7C). However, Δ H-reflex at 40 ms demonstrated a significant and negative correlation with Δ dCOP during the Pre (Figure 7D).

## 4. Discussion

Our findings demonstrate the decreased displacement and velocity of COP in PS3, which indicates that balance performance improved during the third perturbation session. In addition, the observed positive correlation between ∆ MEP (40 ms) and ∆ dCOP (Rec) along with the negative correlation between ∆ H-reflex (40 ms) and ∆ dCOP (Pre) suggests that the decrease in COP displacement may be related to decreased corticospinal excitability and increased spinal excitability. Individual differences were shown in MEPs and H-reflexes from PS1 to PS3, but the neural adaptation for those with improved (eight subjects) balance ability was more likely to transfer from the cortical level toward the spinal level.

The balance performance adaptive process was observed as a sway reduction in both COP displacement and velocity during balance perturbation. In addition, the variability of COP decreased, and COP was close to the standing rest baseline, as shown by the reduction in COP_SD in PS3, indicating body sway. Therefore, balance perturbation training with repeated high amplitude and speed enhanced balance control performance during the third session. This was also in line with the study of Bakker et al. [15], who found that even one session of task-related balance training (but not seated cycling or rest) improved balance performance in specific balance tasks. Several studies have indicated that different types of training improve balance control ability, including sensorimotor training [12], strength training [13], and cognitive dual-task training [14]. Studies also showed that specific balance training improves postural control but not muscle strength in both young and older adults [40,41,42]. The results of our study demonstrated an improvement in balance ability without an increase in muscle strength (i.e., no changes in MVC or EMG activity during the perturbation task were observed), which indicates that the balance task-related training was efficient for improving balance performance in perturbation tasks, at least for young adults (i.e., 28 years old to 42 years old in this study).

During balance perturbation tasks, both feedback control, which occurs in response to sensory feedback, and feedforward control, which refers to the anticipation of a voluntary movement, are involved in postural control [43]. In our previous study [25], balance performance may have been partly learned already following the first testing session, in which dCOP decreased from the Pre to the Rec phase after one perturbation session. It is well accepted that feedforward control is an internal model for accuracy, and does not require feedback loops (e.g., somatosensory feedback), and thus it is more related to anticipation [44]. Therefore, feedforward control might have already improved following the first perturbation session. However, a recent study with a similar setup did not show enhanced balance performance after the first perturbation session [45]. Researchers suggested that balance control ability may not be entirely acquired within just one perturbation session. On the other hand, feedback control relies on information from sensory sources. Since SLR and LLR occur during the Act phase, this study revealed significant balance performance changes in the Act phase during the third session. This suggests that feedback control may play a stronger role in balance control following perturbation training.

The increased MEP amplitude at 40 ms found in our study may be related to the anticipation of the balance system movement, even when masking strategies were in place to reduce the influence of anticipation. To avoid preparatory body position, body swaying was monitored by the perturbation system, and if COP shifted over 5 mm of baseline, perturbation would not be triggered. In the study of Mierau et al. [46], a positive potential (P1) was recorded by electroencephalography (EEG) over the centro-parietal cortical area in the early phase of a balance perturbation, which was suggested to be related to afferent feedback by the perturbation [46]. Therefore, there would not be enough time for the cortical drive to participate in the early perturbation phase. Thus, increasing MEP at 40 ms in our study may be related to feedforward control. In our data, at the late phase of the balance perturbation, an EMG peak after LLR (see Figure 6A) was observed, which can be considered a voluntary muscle activity in order to maintain body position. In the study of Nevanperä et al. [45], a significantly increased V-wave was observed after 70 ms, which indicates that supraspinal drive may be involved in the late phase of the balance perturbation. In the study of Mierau et al. [46], addressing cortical processing of sensory feedback, a negative potential (N1) was recorded with EEG during a 100–200 ms time window after the perturbation onset, which was suggested to be related to the perturbation amplitude and postural threat. The N1 amplitude demonstrated a positive correlation with the EMG activity of the gastrocnemius muscle after 100 ms. A similar positive correlation was observed in our study between EMG activity and MEP in the later phase of the balance perturbation (140 ms). Taken together with the results of Mierau et al. [46], it seems that neural activity at the cortical level is strongly related to muscle activity when trying to maintain balance in the later phases of balance perturbation.

Even though neither MEPs nor H-reflexes demonstrated changes between sessions, the decreased displacement of COP was related to a higher H-reflex but a lower MEP at the 40 ms time onset after the intervention. It seems that neural adaptation to balance perturbation may take place at both the supraspinal and spinal levels. However, the current null findings for changes in MEPs from PS1 to PS3 may also imply that the amount of training was not enough to induce any neural excitability changes cortically and, thus, the movement pattern was not likely fully learned in some subjects (e.g., 3 out of 14 subjects demonstrated increased COP). In a study by Mouthon and Taube [47], increased short-interval intracortical inhibition (SICI) was reported following two weeks (six training sessions) of balance training, showing enhanced intracortical inhibition. In addition, Lauber et al. [48] demonstrated that more cortical inhibition occurred in the initial stages of balance training but then decreased back to baseline as training progressed. Collectively, these findings indicate that there may be a high level cortical drive at the beginning of balance training, which then shifts to a subcortical level once balance control has been acquired [49].

In the current study, only single-pulse TMS at one stimulation intensity was used. Therefore, future studies should consider using paired-pulse TMS to determine intracortical inhibition and facilitation at different delays during balance perturbation tasks. On the other hand, it should also be noted that the adaptation of automaticity-related neural excitability may occur at the spinal level according to modulation of H-reflex to the demands of the task [50]. Previous studies have shown decreasing soleus H-reflex amplitude when human body balance is challenged (i.e., from lying to standing [51], or from standing to walking [52]). In our study, reduced COP indicated that balance control ability was improved after balance perturbation training. In addition, the MEP/H ratio in the majority of subjects (12 out of 14) was lower in PS3 when compared to PS1 (see Figure 5), suggesting that maintaining balance was not as challenging as before. Finally, a study by Bakker et al. [15] found that 30 min balance training improved balance performance, but no neural adaptations such as altered MEP amplitude or increased SICI, as hypothesized, were observed. Researchers speculated that other brain areas, such as the cerebellum, may underlie balance performance improvement [15]. Considering the lack of MEP or H-reflex changes in our study from PS1 to PS3, and the relationship of Δ MEP and Δ H-reflex with Δ dCOP at the 40 ms time point, these findings may imply that subcortical circuit activity could be a crucial factor in balance control, contributing to the enhancement of balance performance in PS3.

Some limitations should be mentioned regarding this study. Large variabilities were observed in both MEP and H-reflex amplitudes when responding to balance perturbation tasks. This variability may have led to the lack of significant differences between sessions. Therefore, more subjects in a future study would be ideal. Despite utilizing a random perturbation order and COP monitor to reduce anticipation and body sway before perturbation onset, it was very difficult to rule out all anticipation influences. Due to a similar order and time interval between the perturbations of each set, it is still possible that subjects could have learned some pattern, which might be one of the reasons for the increasing MEP amplitude at the 40 ms time point.

## 5. Conclusions

Improved balance control, shown by decreased COP displacement and velocity, was observed in session three. However, no significant group-level neural adaptation was shown at the supraspinal or spinal level. A correlation between ∆COP and the ∆MEP/∆H-reflex may imply potential corticospinal excitability changes in parallel with balance performance improvement on an individual level. Based on our findings, it appears that only a few sessions are required to demonstrate motor learning and/or neural adaptation. Although neither the precise driver nor mechanism of adaptation could be identified in the present study, our evidence suggests that repeated short-term balance training led to modifications from cortical to more subcortical functioning.

## Figures and Tables

**Table 1:** dCOP (mm/(m × kg)) and vCOP ((mm/s)/(m × kg)) of Pre, Act, and Rec shown with t-value and *p*-value of the paired-t test. Hedge’s g was used to interpret results with 0.2, 0.5, and 0.8, which were considered as small, medium, and large effects, respectively.

		PS1	PS3	Mean Difference	t _(13)_	*p*-Value	Hedge’s g
dCOP	Pre	0.09 ± 0.04	0.08 ± 0.04	0.01	2.177	0.049 *	0.217
	Act	1.14 ± 0.34	1.01 ± 0.27	0.13	2.483	0.027 *	0.395
	Rec	0.64 ± 0.21	0.44 ± 0.18	0.20	4.642	<0.001 **	0.995
vCOP	Pre	0.16 ± 0.08	0.13 ± 0.06	0.03	2.951	0.011 *	0.429
	Act	1.59 ± 0.57	1.31 ± 0.40	0.28	3.212	0.007 *	0.548
	Rec	0.97 ± 0.35	0.66 ± 0.29	0.31	5.405	<0.001 **	0.928

* (*p* < 0.05); ** (*p* < 0.001).

## Data Availability

All data that support the findings of this study are available upon request according to the GDPR principles. Please contact the corresponding author: Nijia Hu (email: nijia.n.hu@jyu.fi).

## References

[1] Pollock A.S., Durward B.R., Rowe P.J., Paul J.P. (2000). What Is Balance?. Clin. Rehabil..

[2] Horak F.B., Shupert C.L., Dietz V., Horstmann G. (1994). Vestibular and Somatosensory Contributions to Responses to Head and Body Displacements in Stance. Exp. Brain Res..

[3] Jacobs J.V., Horak F.B. (2007). Cortical Control of Postural Responses. J. Neural Transm. Vienna.

[4] Ivanenko Y., Gurfinkel V.S. (2018). Human Postural Control. Front. Neurosci..

[5] Rasman B.G., Forbes P.A., Tisserand R., Blouin J.-S. (2018). Sensorimotor Manipulations of the Balance Control Loop–Beyond Imposed External Perturbations. Front. Neurol..

[6] Petersen N., Christensen L.O.D., Morita H., Sinkjær T., Nielsen J. (1998). Evidence That a Transcortical Pathway Contributes to Stretch Reflexes in the Tibialis Anterior Muscle in Man. J. Physiol..

[7] Taube W., Schubert M., Gruber M., Beck S., Faist M., Gollhofer A. (2006). Direct Corticospinal Pathways Contribute to Neuromuscular Control of Perturbed Stance. J. Appl. Physiol..

[8] Fitzpatrick R.C., Taylor J.L., McCloskey D.I. (1992). Ankle Stiffness of Standing Humans in Response to Imperceptible Perturbation: Reflex and Task-Dependent Components. J. Physiol..

[9] Schieppati M., Nardone A., Siliotto R., Grasso M. (1995). Early and Late Stretch Responses of Human Foot Muscles Induced by Perturbation of Stance. Exp. Brain Res..

[10] Schieppati M., Nardone A. (1997). Medium-Latency Stretch Reflexes of Foot and Leg Muscles Analysed by Cooling the Lower Limb in Standing Humans. J. Physiol..

[11] Kurtzer I.L. (2015). Long-Latency Reflexes Account for Limb Biomechanics through Several Supraspinal Pathways. Front. Integr. Neurosci..

[12] Taube W., Gruber M., Gollhofer A. (2008). Spinal and Supraspinal Adaptations Associated with Balance Training and Their Functional Relevance. Acta Physiol..

[13] Piirainen J.M., Cronin N.J., Avela J., Linnamo V. (2014). Effects of Plyometric and Pneumatic Explosive Strength Training on Neuromuscular Function and Dynamic Balance Control in 60–70 year Old Males. J. Electromyogr. Kinesiol..

[14] Li K.Z., Roudaia E., Lussier M., Bherer L., Leroux A., McKinley P.A. (2010). Benefits of Cognitive Dual-Task Training on Balance Performance in Healthy Older Adults. J. Gerontol. Ser. Biomed. Sci. Med. Sci..

[15] Bakker L.B., Nandi T., Lamoth C.J., Hortobágyi T. (2021). Task Specificity and Neural Adaptations after Balance Learning in Young Adults. Hum. Mov. Sci..

[16] Gruber M., Taube W., Gollhofer A., Beck S., Amtage F., Schubert M. (2007). Training-Specific Adaptations of H- and Stretch Reflexes in Human Soleus Muscle. J. Mot. Behav..

[17] Schubert M., Beck S., Taube W., Amtage F., Faist M., Gruber M. (2008). Balance Training and Ballistic Strength Training Are Associated with Task-Specific Corticospinal Adaptations. Eur. J. Neurosci..

[18] Taube W., Gruber M., Beck S., Faist M., Gollhofer A., Schubert M. (2007). Cortical and Spinal Adaptations Induced by Balance Training: Correlation between Stance Stability and Corticospinal Activation. Acta Physiol..

[19] Correia J.P., Vaz J.R., Domingos C., Freitas S.R. (2022). From Thinking Fast to Moving Fast: Motor Control of Fast Limb Movements in Healthy Individuals. Rev. Neurosci..

[20] Moscatelli F., Messina A., Valenzano A., Monda V., Salerno M., Sessa F., La Torre E., Tafuri D., Scarinci A., Perrella M. (2021). Transcranial Magnetic Stimulation as a Tool to Investigate Motor Cortex Excitability in Sport. Brain Sci..

[21] Rosenkranz K., Kacar A., Rothwell J. (2007). Differential Modulation of Motor Cortical Plasticity and Excitability in Early and Late Phases of Human Motor Learning. J. Neurosci..

[22] Egger S., Wälchli M., Rüeger E., Taube W. (2023). Short-Term Balance Consolidation Relies on the Primary Motor Cortex: A RTMS Study. Sci. Rep..

[23] Prsa M., Thier P. (2011). The Role of the Cerebellum in Saccadic Adaptation as a Window into Neural Mechanisms of Motor Learning. Eur. J. Neurosci..

[24] Krakauer J., Hadjiosif A., Xu J., Wong A., Haith A. (2019). Motor Learning. Comprehensive Physiology.

[25] Hu N., Avela J., Kidgell D.J., Nevanperä S., Walker S., Piirainen J.M. (2022). Reliability of Transcranial Magnetic Stimulation and H-Reflex Measurement during Balance Perturbation Tasks. Front. Physiol..

[26] Freyler K., Gollhofer A., Colin R., Brüderlin U., Ritzmann R. (2015). Reactive Balance Control in Response to Perturbation in Unilateral Stance: Interaction Effects of Direction, Displacement and Velocity on Compensatory Neuromuscular and Kinematic Responses. PLoS ONE.

[27] Pruszynski J.A., Kurtzer I., Scott S.H. (2011). The Long-Latency Reflex Is Composed of at Least Two Functionally Independent Processes. J. Neurophysiol..

[28] Wälchli M., Tokuno C.D., Ruffieux J., Keller M., Taube W. (2017). Preparatory Cortical and Spinal Settings to Counteract Anticipated and Non-Anticipated Perturbations. Neuroscience.

[29] Hermens H., Freriks B., Merletti R., Hägg G., Stegeman D.F., Blok J.H., Hägg G., Blok W.J. (1999). SENIAM 8: European Recommendations for Surface Electromyography.

[30] Blazevich A.J., Kay A.D., Waugh C., Fath F., Miller S., Cannavan D. (2012). Plantarflexor Stretch Training Increases Reciprocal Inhibition Measured during Voluntary Dorsiflexion. J. Neurophysiol..

[31] Kirk B.J.C., Trajano G.S., Pulverenti T.S., Rowe G., Blazevich A.J. (2019). Neuromuscular Factors Contributing to Reductions in Muscle Force After Repeated, High-Intensity Muscular Efforts. Front. Physiol..

[32] Rodriguez-Falces J., Place N. (2018). End-of-Fiber Signals Strongly Influence the First and Second Phases of the M Wave in the Vastus Lateralis: Implications for the Study of Muscle Excitability. Front. Physiol..

[33] Piirainen J.M., Linnamo V., Cronin N.J., Avela J. (2013). Age-Related Neuromuscular Function and Dynamic Balance Control During Slow and Fast Balance Perturbations. J. Neurophysiol..

[34] Sen C.B.A., Fassett H.J., El-Sayes J., Turco C.V., Hameer M.M., Nelson A.J. (2017). Active and Resting Motor Threshold Are Efficiently Obtained with Adaptive Threshold Hunting. PLoS ONE.

[35] Groppa S., Oliviero A., Eisen A., Quartarone A., Cohen L.G., Mall V., Kaelin-Lang A., Mima T., Rossi S., Thickbroom G.W. (2012). A Practical Guide to Diagnostic Transcranial Magnetic Stimulation: Report of an IFCN Committee. Clin. Neurophysiol..

[36] Chiari L., Rocchi L., Cappello A. (2002). Stabilometric Parameters Are Affected by Anthropometry and Foot Placement. Clin. Biomech..

[37] Hirano M., Kubota S., Koizume Y., Tanaka S., Funase K. (2016). Different Effects of Implicit and Explicit Motor Sequence Learning on Latency of Motor Evoked Potential Evoked by Transcranial Magnetic Stimulation on the Primary Motor Cortex. Front. Hum. Neurosci..

[38] Avenanti A., Paluello I.M., Bufalari I., Aglioti S.M. (2006). Stimulus-Driven Modulation of Motor-Evoked Potentials during Observation of Others’ Pain. NeuroImage.

[39] Nielsen J.F. (1996). Logarithmic Distribution of Amplitudes of Compound Muscle Action Potentials Evoked by Transcranial Magnetic Stimulation. J. Clin. Neurophysiol..

[40] Buchner D.M., Cress M.E., De Lateur B.J., Esselman P.C., Margherita A.J., Price R., Wagner E.H. (1997). The Effect of Strength and Endurance Training on Gait, Balance, Fall Risk, and Health Services Use in Community-Living Older Adults. J. Gerontol. A Biol. Sci. Med. Sci..

[41] Howe T.E., Rochester L., Neil F., Skelton D.A., Ballinger C. (2011). Exercise for Improving Balance in Older People. Cochrane Database Syst. Rev..

[42] Kurz I., Gimmon Y., Shapiro A., Debi R., Snir Y., Melzer I. (2016). Unexpected Perturbations Training Improves Balance Control and Voluntary Stepping Times in Older Adults—A Double Blind Randomized Control Trial. BMC Geriatr..

[43] Dietz V., Trippel M., Ibrahim I.K., Berger W. (1993). Human Stance on a Sinusoidally Translating Platform: Balance Control by Feedforward and Feedback Mechanisms. Exp. Brain Res..

[44] Kawato M. (1999). Internal Models for Motor Control and Trajectory Planning. Curr. Opin. Neurobiol..

[45] Nevanperä S., Hu N., Walker S., Avela J., Piirainen J.M. (2023). Modulation of H-Reflex and V-Wave Responses during Dynamic Balance Perturbations. Exp. Brain Res..

[46] Mierau A., Hülsdünker T., Strüder H.K. (2015). Changes in Cortical Activity Associated with Adaptive Behavior during Repeated Balance Perturbation of Unpredictable Timing. Front. Behav. Neurosci..

[47] Mouthon A., Taube W. (2019). Intracortical Inhibition Increases during Postural Task Execution in Response to Balance Training. Neuroscience.

[48] Lauber B., Gollhofer A., Taube W. (2021). What to Train First: Balance or Explosive Strength? Impact on Performance and Intracortical Inhibition. Scand. J. Med. Sci. Sports.

[49] Logan G.D. (1979). On the Use of a Concurrent Memory Load to Measure Attention and Automaticity. J. Exp. Psychol. Hum. Percept. Perform..

[50] Adkins D.L., Boychuk J., Remple M.S., Kleim J.A. (2006). Motor Training Induces Experience-Specific Patterns of Plasticity across Motor Cortex and Spinal Cord. J. Appl. Physiol..

[51] Mynark R.G., Koceja D.M. (2002). Down Training of the Elderly Soleus H Reflex with the Use of a Spinally Induced Balance Perturbation. J. Appl. Physiol..

[52] Capaday C., Stein R.B. (1986). Amplitude Modulation of the Soleus H-Reflex in the Human during Walking and Standing. J. Neurosci..

